# Antivascular Therapy for Epithelial Ovarian Cancer

**DOI:** 10.1155/2010/372547

**Published:** 2009-12-23

**Authors:** Francois P. Duhoux, Jean-Pascal Machiels

**Affiliations:** ^1^Centre du Cancer, Department of Medical Oncology, Cliniques Universitaires Saint-Luc, Université Catholique de Louvain, 1200 Brussels, Belgium; ^2^F.R.S.-FNRS Research Fellow, Belgium

## Abstract

Ovarian cancer is the fifth largest cancer killer in women. Improved understanding of the molecular pathways implicated in the pathogenesis of ovarian cancer has led to the investigation of novel targeted therapies. Ovarian cancer is characterized by an imbalance between pro- and antiangiogenic factors in favor of angiogenesis activation. Various antivascular strategies are currently under investigation in ovarian cancer. They can schematically be divided into antiangiogenic and vascular-disrupting therapies. This paper provides a comprehensive review of these new treatments targeting the tumor vasculature in this disease. Promising activities have been detected in phase II trials, and results of phase III clinical trials are awaited eagerly.

## 1. Introduction

Ovarian cancer is the fifth largest cancer killer in women. Primary surgical cytoreduction followed by platinum-based chemotherapy is the standard treatment for patients with advanced epithelial ovarian cancer. However, despite this aggressive approach, all stages combined, the 5-year survival rate remains only around 45% [1]. Novel approaches to improve disease outcome are thus urgently needed.

**Figure 1:**
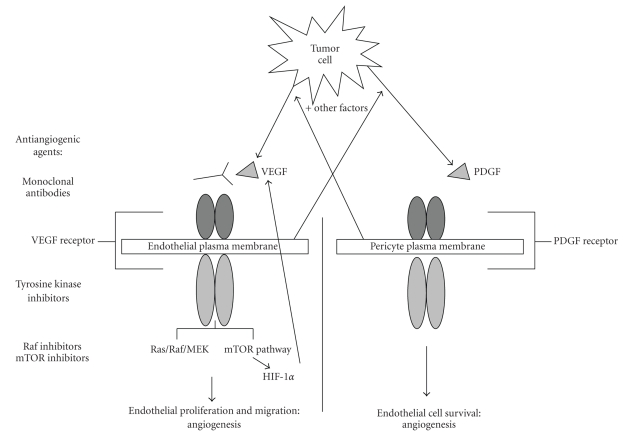
Major pathways promoting angiogenesis in epithelial ovarian cancer. VEGF: vascular endothelial growth factor, PDGF: platelet-derived growth factor, mTOR: mammalian target of rapamycin.

**Figure 2:**
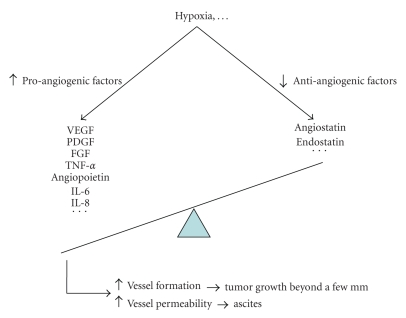
Molecular events leading to increased angiogenesis in epithelial ovarian cancer. VEGF: vascular endothelial growth factor, PDGF: platelet-derived growth factor, FGF: fibroblast growth factor, TNF = tumor necrosis factor, IL: interleukin.

There is a strong rationale to use antivascular therapies in epithelial ovarian cancer. Ovarian cancer is characterized by an imbalance between pro- and antiangiogenic factors in favor of angiogenesis activation, with an increase in the tumor levels of proangiogenic factors (i.e., vascular endothelial growth factor (VEGF), fibroblast growth factor (FGF), platelet-derived growth factors (PDGFs), tumor necrosis factor (TNF)-alpha, angiopoietins, interleukin (IL-6 and IL-8, etc.) and a decrease in anti-angiogenic factors (i.e., angiostatins, endostatins, etc.) [2]. Angiogenesis is necessary for tumors to grow beyond a few millimeters and is triggered by tumor hypoxia that induces the release of pro-angiogenic factors [3]. Angiogenesis has also an important role in the formation of ascites, a frequent clinical feature of advanced ovarian cancer. The accumulation of ascites results mainly from the increased permeability of the peritoneal capillaries. VEGF, also known as the “vascular permeability factor,” plays a key role in this process [4] (see Figures 1 and 2).

Various antivascular strategies have been investigated in ovarian cancer. They can schematically be divided into antiangiogenic therapies and vascular-disrupting therapies. Given the important role of vascular biology in ovarian cancer, it is not surprising that these new treatment approaches have shown promising activity in this disease, even when administered as a single agent.

## 2. Antiangiogenic Therapies

### 2.1. VEGF

The most studied antiangiogenic strategies target the VEGF/VEGF receptor (VEGFR) pathway through inhibition of its ligands and/or receptors. The VEGF family includes 6 glycoproteins (VEGF-A to E and placental growth factor) and 3 tyrosine kinase receptors (VEGFR1 to 3). VEGF-A promotes angiogenesis through enhancement of permeability, activation, survival, migration, invasion, and proliferation of endothelial cells [5]. VEGFR1 and VEGFR2 mediate the effects of VEGF-A [6]. Recent studies suggest a direct effect of VEGF-A on tumor cell proliferation the VEGFR2 via a mechanism thought to involve the AKT/mTOR pathway [7]. VEGF-A also regulates the invasiveness of cancer cells by altering the expression of matrix metalloproteinase-2 [8].

#### 2.1.1. Agents Directed Against VEGF Ligand(S)

(1) The most widely investigated anti-VEGF ligand agent is *bevacizumab (BEV)*. BEV is a recombinant humanized monoclonal antibody that binds and neutralizes all biologically active isoforms of VEGF. Published studies are presented in this section, while ongoing trials are summarized in Table 1.

**(a) Single-Agent Activity**

In 2005, Monk et al. reported an objective response lasting more than 5 months in a patient treated with BEV monotherapy after failing eleven lines of chemotherapy and radiation therapies [9]. Later, the same group found a 16% objective response rate (ORR) in a retrospective analysis of 32 patients with refractory epithelial ovarian cancer treated with BEV alone or in combination with chemotherapy (after failing 2 to 10 prior cytotoxic regimens) [10].

In the phase II GOG 170-D trial, Burger et al. reported a partial response (PR) rate of 18% (11 out of 62) and a complete response (CR) rate of 3% (2 out of 62) in patients with persistent or recurrent epithelial ovarian cancer or primary peritoneal cancer having received 1 or 2 prior cytotoxic regimens and treated with BEV monotherapy. Median progression-free survival (PFS) was 4.7 months [11]. These results were confirmed by Cannistra et al. who observed PR in 15.9% (7 out of 44) with a median PFS of 4.4 months with single-agent BEV in women with refractory or resistant ovarian cancer or peritoneal serous cancer [12].

BEV maintenance therapy after complete response to cisplatin-based chemotherapy is an interesting concept and showed promising results in xenograft models of ovarian cancer by prolonging survival [13]. This approach is currently explored in scheduled and ongoing trials (see Table 1).

**(b) BEV and Chemotherapy**

The vessels formed during tumor angiogenesis are structurally and functionally abnormal. This leads to an impaired tumor blood supply that may interfere with the delivery of therapeutics. Hypoxia also renders tumor cells more resistant to both radiation and cytotoxic drugs [14]. It has been proposed that the “normalization” of the tumor vasculature by BEV could allow a better delivery of chemotherapy and decrease hypoxia, making tumors more chemosensitive [15].

In a retrospective analysis of 23 patients with recurrent platinum-refractory epithelial ovarian cancer progressing after 2 to 15 prior cytotoxic regimens, Wright et al. observed PR in 35% with a combination of BEV associated with various chemotherapy regimens (cyclophosphamide, 5-fluorouracil, docetaxel, or gemcitabine/liposomal doxorubicin). Median PFS was 5.6 months in the patients who achieved a PR [16]. Richardson et al. reported an ORR of 78% and a median PFS of 12 months in a retrospective analysis of 35 patients with recurrent ovarian cancer treated with a combination of gemcitabine, cisplatin or carboplatin and BEV. The higher ORR observed in this last study could be explained by the inclusion of a vast majority of platinum-sensitive patients [17]. Cohn et al. retrospectively identified 10 patients with advanced, recurrent and refractory ovarian cancer who were treated with a combination of BEV and weekly taxane (paclitaxel or docetaxel) after failure of 1 to 4 prior chemotherapy regimens. The 5 symptomatic patients in this study experienced a rapid subjective palliation of pain, nausea, and ascites [18].

Metronomic administration of chemotherapy, defined as the frequent administration of doses substantially lower than the maximum tolerated dose, can suppress tumor growth, probably through stimulation of the release of thrombospondin 1, a potent and endothelial-specific inhibitor of angiogenesis [19]. Shortening the time between cycles provides more sustained apoptosis of endothelial cells within the tumor vascular bed [20]. Metronomic chemotherapy regimens deliver lower doses of cytotoxic agents, thereby decreasing potential side effects and improving patient tolerance [21]. In a retrospective analysis, 15 heavily pretreated patients with recurrent ovarian cancer (5–15 prior chemotherapy regimens) received a combination of BEV and metronomic oral cyclophosphamide with encouraging results: CR 13.3% and PR 40% [22]. However, in a prospective phase II trial that included 70 less heavily pretreated patients with recurrent ovarian cancer, combination of BEV with metronomic cyclophosphamide showed PR in only 24% with a median PFS of 7.2 months [23].

Various combinations of BEV and chemotherapy are currently being *tested*; these studies are briefly described in Table 1.

**(c) BEV and Other Targeted Therapies**

Combination therapy in this context can be divided into horizontal and vertical molecular pathway blockade. The horizontal approach involves the association of targeted agents to inhibit two or more different pathways simultaneously, while the vertical approach involves the inhibition of various molecular steps of the same pathway, thus counteracting negative feedback loops. By inhibiting the activation of alternate molecular pathways, these combinations could theoretically decrease treatment resistance [24].

Epithelial growth factor receptor (EGFR) is overexpressed in up to 70% of advanced epithelial ovarian cancers [25] and an increased level of EGFR expression has been correlated with poorer overall survival [26]. The VEGF and the EGFR pathways are interconnected: VEGF signaling is upregulated by EGFR expression and VEGF upregulation independent of EGFR signaling seems to contribute to resistance to EGFR inhibition [27]. Since EGFR inhibitors alone have shown limited activity in epithelial ovarian cancer [28, 29], it was postulated that combining an EGFR tyrosine kinase inhibitor like erlotinib with BEV might improve response rates. Unfortunately, in a phase II trial conducted in 13 patients with recurrent ovarian cancer treated with a combination of BEV and erlotinib after failure of 1 to 3 prior chemotherapy regimens, ORR was relatively low (15%) and median PFS (4.1 months) did not seem to be improved over BEV alone [30]. Other trials investigating this combination are planned (see Table 1).

Sorafenib inhibits, among others, the VEGFR2 and Raf kinases. In a phase I dose-escalation study with a combination of BEV and sorafenib, 6 of 13 (46%) patients with ovarian cancer had a PR [31]. A phase II study with this combination is ongoing (see Table 1).

Other combinations of BEV with targeted therapies have been tested in preclinical models. Since the mammalian target of rapamycin (mTOR) pathway regulates VEGF expression in cancer cells [32], researchers combined BEV and rapamycin in an ovarian cancer xenograft model and found a 94% reduction in tumor growth as well as a prolonged survival [33]. Everolimus, another mTOR inhibitor, is now under investigation in a randomized phase II trial (see Table 1).

**(d) Toxicities of BEV**

Angiogenesis inhibitors are not easy drugs to manipulate with some specific toxicities. Common *complications* following treatment with BEV in colorectal cancer, where this drug is widely used, include hypertension (25% grade 1-2, 5% grade 3-4), proteinuria (9% grade 1-2, 1% grade 3-4), bleeding (28% grade 1-2, 3% grade 3-4), wound-healing complications (3% grade 1-2, 1% grade 3-4), arterial thrombo-embolic events (1.5%, mostly grade 3-4), and gastrointestinal (GI) perforations (2%, mostly grade 3-4, with only 0.4% grade 5) [34]. The complication rate in ovarian cancer is quite similar, but there are some noteworthy specificities. In the published phase II ovarian studies, the rate of GI perforations varied from 0% [11] to 11.4% [12], leading to the early closure of the latter study. It was hypothesized that the increased rate of bowel perforation in the latter study was due to the fact that these patients were more heavily pretreated, but this finding could not be confirmed in other studies. Intestinal obstruction and bowel wall involvement by the tumor were other potential risk factors, but they were not statistically significant. In a retrospective review of 62 patients treated with BEV after a median of 5 prior chemotherapy regimens, researchers found grade 3–5 toxicities in 24% of patients, including grade 3-4 hypertension in 7%, GI perforations in 7%, and chylous ascites (probably due to lymphatic disruption by targeting VEGF-C) in 5%. Development of GI perforations and chylous ascites appeared to correlate with tumor response [35].

There is a trend towards increased toxicity when BEV is combined with a cytotoxic agent [35]. GI perforation seems to be more frequent in ovarian cancer than in other solid tumors and could be favored by peritoneal carcinomatosis. In a retrospective cohort of patients without clinical symptoms of bowel obstruction and without evidence of bowel involvement, there were no cases of GI perforation or other grade 3/4 toxicities [36]. Careful patient selection might reduce the risk of GI perforations but all toxicities will not be avoided. Researchers recently reported two cases of GI perforations in a retrospective analysis of 35 patients treated with gemcitabine, platinum, and BEV. These patients had none of the abovementioned risk factors and were not heavily pretreated [17]. It seems in any case preferable to withhold therapy for at least 30 days before surgery [37].

Rare complications reported specifically in ovarian cancer patients treated with BEV include spontaneous nasal septal perforation [38] and erosive osteoarthritis [39].

(2) Aflibercept (VEGF-trap) is a VEGF-ligand-binding antiangiogenic agent that binds and inactivates VEGF-B and placental growth factor in addition to VEGF-A. In preclinical models of ovarian cancer, it significantly reduced both tumor burden and ascites [40, 41]. A phase I trial of VEGF-trap in patients with advanced solid tumors included one patient with ovarian cancer. This patient experienced a PR. Fatigue (9 out of 10 patients), pain (4 out of 10 patients), and constipation (4 out of 10 patients) were the most common side effects of this new drug [42]. In a phase II trial of VEGF-trap in patients with platinum-resistant and topotecan and/or liposomal doxorubicin-resistant advanced ovarian cancer [43] an interim analysis after accrual of 162 patients showed that 11% of the patients receiving the study drug experienced a PR [44]. A phase II trial of VEGF-trap in advanced ovarian cancer patients with recurrent symptomatic malignant ascites [45] has been completed but not yet reported. A phase II trial combining VEGF-trap with docetaxel in patients with persistent or recurrent ovarian epithelial cancer is currently ongoing [46].

(3) *HuMV833* is another monoclonal antibody directed against VEGF. In a phase I study conducted in patients with advanced cancer, one patient with ovarian cancer experienced a PR that lasted 9 months [47].

#### 2.1.2. VEGFR Tyrosine Kinase Inhibitors

*Cediranib* (AZD2171, CED) is a highly selective and potent oral tyrosine kinase inhibitor (TKI) of VEGFR1, VEGFR2, VEGFR3, and c-Kit. In a phase II study conducted in recurrent epithelial ovarian cancer, researchers found CED to have an ORR of 18.5%. Grade 3 toxicities included hypertension (13 out of 27 patients), fatigue (5 out of 27 patients), diarrhea (3 out of 27 patients), vomiting (2 out of 27 patients), hyponatremia (2 out of 27 patients), oral cavity pain (2 out of 27 patients), and nausea, constipation, abdominal pain, headache, and hypothyroidism (1 out of 27 patients). Grade 4 toxicities included central nervous system hemorrhage (1 out of 27 patients), lipase elevation (1 out of 27 patients), and hypertriglyceridemia (1 out of 27 patients) [48]. Two phase II trials are currently studying CED in recurrent ovarian cancer [49, 50], while a phase III randomized study is comparing chemotherapy with carboplatin and paclitaxel (PBC) versus concurrent CED and PBC versus concurrent CED and PBC followed by maintenance CED in women with platinum-sensitive relapsing ovarian epithelial carcinoma [51].

*Ramucirumab *(IMC-1121B) is a fully human antibody that blocks the interaction between VEGF and VEGFR2, resulting in potent inhibition of an array of biological activities of VEGF, including activation of the receptor and its signaling pathway, intracellular calcium mobilization, and migration and proliferation of endothelial cells [52]. It is currently under study in a phase II trial of persistent or recurrent epithelial ovarian carcinoma [53].

*Semaxinib* (SU5416) is a tyrosine kinase inhibitor with activity against VEGFR2. It reduced microvessel density and tumor growth in a preclinical tumor model with high VEGF expression [54].

Despite these promising data, some combination trials resulted in very disappointing results. In a recent preclinical study of metronomic paclitaxel with the VEGFR2 inhibitor SU5416, researchers found that the combination therapy showed an additive effect in tumors with low VEGF expression, while they observed an antagonism in tumors with high VEGF expression. They postulated that the lack of additive effect between these 2 drugs in tumors with high VEGF expression might be due to the fact that these two agents acted through the same pathways, and that their concomitant use could not produce more effects than each drug used in monotherapy [54]. These experiments outline that a better knowledge of the various molecular pathways implicated will help us to investigate the optimal combination partners and schedules.

### 2.2. PDGF

Platelet-derived growth factor (PDGF) is a potent mitogen and chemotactic factor for a variety of mesenchymal cells, such as fibroblasts and vascular smooth muscle cells. They exert their effects on target cells by activating two structurally related protein tyrosine-kinase receptors, *α* and *β* located on pericytes [55]. High expression of PDGF receptors is a common characteristic of solid tumors [56].

PDGF is expressed in 73% of ovarian carcinomas, while 36% express PDGF-receptor alpha (PDGFRA). In addition, overexpression of PDGFRA is an independent poor prognostic factor in ovarian carcinoma [57]. *Imatinib* mesylate is a small molecule that inhibits the tyrosine kinases abl, c-kit, PDGFRA, and PDGFRB. It inhibits the growth of ovarian cancer cells through PDGFRA inactivation [58], and decreases the secretion of VEGF by epithelial ovarian cancer cells [59]. However, in the clinical setting, imatinib has failed to show relevant clinical activity as a *single* agent. There was no complete or partial response with imatinib monotherapy in a phase II trial that enrolled 16 patients with platinum/taxane-resistant disease overexpressing at least one imatinib molecular target [60]. In another phase II trial with imatinib in a less pretreated ovarian cancer population, median PFS was also disappointingly low: 2 months [61]. There are various reasons for the ineffectiveness of imatinib monotherapy in ovarian cancer: downregulation of c-kit and PDGFR may lead to induction of VEGF, inhibition of a single tyrosine kinase might be insufficient to impact downstream signaling cascades, and the molecular targets of imatinib might not be relevant in the occurrence of ovarian cancer in comparison with gastrointestinal stromal tumor or chronic myeloid leukemia where a single specific mutation or a translocation, respectively, can be responsible for the genesis of these two cancers [62]. Despite these results, a phase II study of imatinib monotherapy in patients with recurrent platinum and taxane-resistant epithelial ovarian cancer whose tumor expresses either c-kit, PDGFR, or ABL is currently accruing patients [63].

By dysregulating proangiogenic signaling, there was some hope that the use of imatinib in a *combination* approach might be more effective. This was supported by a preclinical model of human ovarian carcinoma in which combination treatment with imatinib and paclitaxel induced increased apoptosis of tumor-associated endothelial cells, which resulted in a reduced tumor burden [64]. However, combination therapy with imatinib and docetaxel in 23 heavily pretreated patients with advanced, platinum-resistant ovarian cancer, and primary peritoneal carcinomatosis resulted in a disappointing ORR of 21.7% (1 CR and 4 PR) and a median PFS of 1.8 months [65]. A phase II study is currently studying the combination of paclitaxel with imatinib in taxane-pretreated ovarian cancer [66].

### 2.3. Multitargeted Tyrosine Kinase Inhibitors

Targeting the PDGF/PDGFR axis alone or in combination with classical chemotherapy is not very effective in the clinical setting. Endothelium homeostasis is regulated to a large extent by the PDGF/PDGFR system expressed by pericytes. Pericytes are perivascular cells that provide local survival signals for endothelial cells. Combination approaches targeting the VEGF/VEGFR and the PDGF/PDGFR axes are thus very appealing [67].

*Sunitinib (SUN)* is an orally bioavailable small molecule that inhibits multiple tyrosine kinases including all the PDGF receptors and VEGF receptors, as well as c-kit, RET, CSF-1R, and flt-3. A patient with recurrent clear cell ovarian carcinoma briefly responded to SUN as fifth-line therapy [68]. At least three phase II trials of SUN in recurrent and refractory ovarian carcinoma are currently ongoing (see Table 2). Typical side effects of SUN in other diseases are fatigue (28% grade 2-3), diarrhea (20% grade 2-3), dyspepsia (16% grade 2-3), hypertension (16% grade 2-3), hand-foot syndrome (15% grade 2-3), nausea (13% grade 2), stomatitis (13% grade 2-3), anorexia (12% grade 2-3), neutropenia (40% grade 2-3, 2% grade 4), thrombocytopenia (21% grade 2-3), lipase elevations (25% grade 2-3, 3% grade 4) [69], and hypothyroidism (53–85%) [70].

*Sorafenib (SOR)* is an oral small molecule that predominantly inhibits the serine/threonine raf-1 kinase. The molecule also inhibits other tyrosine kinase receptors including VEGFR1, VEGFR2, VEGFR3, PDGFRB, flt-3, and c-kit. The Ras/raf/MEK kinase pathway plays a key role in cellular proliferation. In addition, the Raf kinase is a downstream modulator of the VEGF signaling pathway [71]. Oncogenic b-raf mutations have been found with high frequency in ovarian cancer [72, 73]. After encouraging phase I results, where about 50% of patients with epithelial ovarian cancer had evidence of stable disease [74], SOR is now being tested in various combinations (see Table 2). 

*Vatalanib* is a multitargeted tyrosine kinase inhibitor targeting angiogenesis that inhibits PDGFRB, VEGFR1, VEGFR2, c-Kit, and c-Fms. In a preclinical model of VEGF-dependent human ovarian carcinomas, vatalanib inhibited the formation of malignant ascites and the tumor growth [75, 76]. It is currently under investigation in advanced solid tumors. 

*BIBF 1200* is a combined inhibitor of PDGFR, VEGFR, and FGFR [77]. It was tested as maintenance therapy in a phase II randomized double-blind trial in ovarian cancer patients who responded to their last (at least second line) chemotherapy. Median time to RECIST progression was 4.8 months for BIBF 1120, and 2.8 months for placebo. Grade 3 and 4 adverse events were seen in 54 and 7% (BIBF 1120) and 25 and 3% (placebo) of patients. The rate of gastrointestinal toxicities was slightly higher in the BIBF 1120 arm (16 versus 10%, all grade 3; no grade 4 events). Elevation of liver enzymes occurred in 43% (BIBF 1120) versus 6.3% (placebo) [78]. *Other multitargeted tyrosine kinase inhibitors currently under investigation are summarized in Table 2.*

### 2.4. Endothelin

The *endothelin* axis comprises 3 small peptides (ET-1 to -3) that mediate various physiological processes by binding to endothelin A (ET_A_) and endothelin B (ET_B_) surface receptors. Activation of the ET_A_ receptor (ET_A_R) by ET-1 increases tumor cell proliferation, survival, angiogenesis, migration, invasion, and metastasis in ovarian cancer [79]. Endothelins also modulate angiogenesis indirectly, as VEGF and ET-1 have reciprocal stimulatory interactions in vivo [80]. More than 90% of primary ovarian cancers express ET-1, and ET-1 expression in tumors is significantly elevated compared to normal ovarian tissue. Moreover, the vast majority of ovarian carcinomas express the ET_A_R [81], which is emerging as an attractive target for anti-angiogenesis therapy. 

Atrasentan is a selective ET_A_R antagonist. In ovarian carcinoma xenografts, atrasentan significantly reduced microvessel density, expression of VEGF, matrix metalloproteinase-2, and increased the percentage of apoptotic tumor cells. Combined treatment with atrasentan and paclitaxel produced additive antitumor, apoptotic, and antiangiogenic effects [82]. 

In humans, the most common side effects of atrasentan include fatigue, edema, and rhinitis [83].

In a preclinical model, ZD4054, another selective ET_A_R antagonist, significantly reduced tumor growth and angiogenesis [84]. The reduction in new vessel formation was even more pronounced when ZD4054 was combined with gefitinib [85]. As is the case with atrasentan, the combination of ZD4054 with paclitaxel also produced additive antitumor effects [86].

### 2.5. mTOR Inhibitors

Inhibition of mTOR reduces secretion of VEGF by the tumor through inhibition of HIF-1*α*. In addition, mTOR inhibitors can also decrease cancer cell proliferation and survival [87]. RAD001 (everolimus) diminished the expression of VEGF and inhibited angiogenesis in a transgenic mouse model of ovarian cancer [88]. RAD001 significantly enhanced cisplatin-induced apoptosis in vitro [89]. A randomized phase II study of BEV with or without everolimus in patients with recurrent or persistent ovarian epithelial cancer is ongoing [90].

### 2.6. Src Inhibition

Src plays a critical role in tumor angiogenesis, probably through the regulation of IL-8, an important angiogenic cytokine [91–93]. It is also essential for the hypoxia-mediated induction of VEGF [94]. Src inhibition through a novel small-molecule inhibitor, AP23994, alone or in combination with cytotoxic chemotherapy, significantly reduced tumor growth in ovarian cancer models [95]. Src is thus emerging as a new target for antiangiogenic treatment of ovarian cancer. A phase I trial of a Src kinase inhibitor, dasatinib, in combination with paclitaxel and carboplatin in patients with advanced or recurrent ovarian cancer is currently ongoing [96]. Src inhibition is also being evaluated in a phase I study combining dasatinib and BEV in patients with metastatic or unresectable solid tumors [97].

AZD0530 is a dual inhibitor of Src and abl. It is currently in phase II study in combination with carboplatin plus paclitaxel in platinum-sensitive ovarian cancer patients [98].

EphA2 is a protein overexpressed by many tumor cells. Use of an agonistic antibody of EphA2 (EA5) in combination with paclitaxel substantially reduced tumor growth in an ovarian cancer model, including a paclitaxel-resistant model. EA5 led to dissociation of Src from EphA2, resulting in decreased phosphorylation of Src and thus VEGF expression [99].

### 2.7. Integrin *α*5*β*1 Targeting

Endostatin is a COOH-terminal fragment of collagen XVIII and is a potent angiogenesis inhibitor. Integrin *α*5*β*1 is the major target for endostatin-mediated inhibition of endothelial cell proliferation and migration. Endostatin was shown to block peritoneal attachment and vessel cooption by ovarian cancer cells [100]. It is currently being investigated in phase I studies in advanced refractory solid tumors [101, 102]. Volociximab is a chimeric monoclonal antibody that blocks *α*5*β*1 binding to fibronectin and induces apoptosis in proliferating endothelial cells. It was tested in a phase I/II study in combination with pegylated doxorubicin in patients with recurrent platinum-resistant ovarian cancer. Since a preliminary analysis of PFS suggested that there was a low probability of detecting a statistically significant difference in favor of the combination regimen, the study was closed to enrollment [103].

### 2.8. Thalidomide (THAL)

Multiple mechanisms of action have been proposed for THAL. It could, at least in part, act through an antiangiogenic effect, by inhibiting tumor-necrosis alpha, VEGF and/or fibroblast growth factor 2 [104]. In a phase I study involving 17 heavily pretreated patients with recurrent epithelial ovarian cancer, 18% experienced a PR and 35% a stable disease after 6 months. Median time to progression was 10 months. Common grade 1 or 2 side effects included constipation (76%), neuropathy (71%) and fatigue (65%). Among the 5 grade 3/4 toxicities, 2 patients (12%) had a venous thrombosis [105]. A single-institution prospective cohort study conducted in patients with recurrent ovarian or primary peritoneal cancer who had received a minimum of 2 prior therapeutic regimens compared any standard intravenous chemotherapy to THAL or treatment holiday. There was a trend towards comparable responses in the chemotherapy and THAL arms. There was a high rate of grade 3 dyspnea, with 8 out of 18 (44%) patients who presented subjective shortness of breath at rest in the THAL arm. At least one of these patients had pulmonary embolus, a dreaded complication of THAL [106]. In a randomized phase 2 trial comparing topotecan to topotecan plus THAL in 75 women with recurrent epithelial ovarian cancer, the addition of THAL to topotecan appeared to improve response rates: ORR was 47% in the THAL arm versus 21% in the topotecan alone arm. Median PFS was 6 months in the THAL arm compared to 4 months in the control arm [107]. A randomized phase II study is currently comparing carboplatin and THAL with carboplatin alone in patients with stage Ic-IV ovarian cancer [108].

### 2.9. Prostaglandin E2 (PGE2)

PGE2 enhances angiogenesis through the induction of VEGF [109]. Clofibric acid is a peroxysome proliferator-activated receptor *α* (PPAR*α*) ligand that reduces PGE2 levels, leading to repression of VEGF expression, inhibition of angiogenesis and tumor cell apoptosis in a preclinical ovarian cancer model [110]. In a preclinical ovarian cancer model, celecoxib, a cyclooxygenase-2 (COX-2) inhibitor, and ciglitazone, a PPAR*γ* ligand, reduced tumor growth by decreasing angiogenesis through inhibited VEGF production in relation to PGE2 reduction [111]. Ongoing trials are investigating celecoxib in advanced ovarian cancer; one phase II study is combining paclitaxel with celecoxib [112] and another randomized phase II study is comparing cyclophosphamide with or without celecoxib [113].

### 2.10. Antiangiogenic Gene Therapy

(i) Phosphatase and tensin homologue on chromosome 10 (PTEN) is a cancer suppressor gene. Overexpression of the PTEN gene by transfection in ovarian cancer cell lines without PTEN mutations leads to decreased VEGF concentrations and a reduced number of new blood vessels. PTEN gene therapy in murine models of human ovarian cancer suppresses intraperitoneal dissemination and extends survival [114].

(ii) Increased IL-8 expression is associated with poor clinical outcome in human ovarian carcinoma, and IL-8 gene silencing with small interfering RNAs (siRNAs) can decrease tumor growth through antiangiogenic mechanisms in preclinical models [115].

(iii) Ribozymes are catalytic RNA molecules that can cleave other RNA molecules in a target-specific manner, thereby downregulating the expression of any pathogenic gene product. Angiozyme inhibits angiogenesis by selectively downregulating VEGFR1 through targeted cleavage of VEGFR1 mRNA [116]. After encouraging phase I testing, it has now completed the phase II setting in renal cancer [117]. There is a strong rationale to try this approach in ovarian cancer.

(iv) Shiga-like toxin 1 mutants Stx1^W203F^ and Stx1^R170H^ have been shown in preclinical models to have antiproliferative and antiangiogenic effects in murine xenograft models of ovarian cancer. They are good candidates for gene therapy [118].

### 2.11. Other Antiangiogenic Targets

(i) Squalamine is an aminosterol that inhibits mitogen-induced proliferation and migration of endothelial cells in vitro and causes significant in vivo inhibition of angiogenesis [119]. It is currently in phase II testing in combination with carboplatin in patients with recurrent or refractory stage III or stage IV ovarian cancer [120].

(ii) CAI is a synthetic carboxyamidotriazole that inhibits proliferation, invasion and metastasis, and neovascularization both in vitro and in vivo. In a phase II study of 38 heavily pretreated patients with recurrent epithelial ovarian cancer, median PFS was 3.6 months [121].

(iii) Angiopoietins are emerging as crucial regulators of the angiogenic switch in tumors [122]. AMG 386 is a peptibody that binds to and inhibits angiopoietin 1 and 2. It is being investigated in a phase 1b study in combination with either pegylated liposomal doxorubicin or topotecan in subjects with advanced recurrent epithelial ovarian cancer [123].

## 3. Vascular-Disrupting Agents

Tumor vessels have different characteristics than normal vessels. They have been found to be more tortuous, less organized, and more leaky [124]. Vascular-disrupting agents (VDAs) are a new class of agents that cause a pronounced shutdown in blood flow to solid tumors, resulting in extensive tumor-cell necrosis due to lack of oxygen and nutrients supply, while they leave the blood flow in normal tissues relatively intact [125]. Small molecules VDAs are the major class of VDAs. They can be divided into 2 groups: the tubulin-binding agents and the flavonoids [126].

Combretastatin A-4 (*CA-4*), its prodrug ZD6126 and AVE8062 (a water-soluble analog of CA-4) are tubulin-binding agents that are structurally related to the colchicines and possess potent antivascular properties [126]. CA-4 was shown to exert its antivascular effects through selective disruption of the tubulin cytoskeleton of endothelial cells [127]. In a murine model of ovarian carcinoma, AVE8062 effectively inhibited tumor growth and was even more effective in combination with docetaxel [128]. VDAs are currently in clinical development, alone or in combination. 5, 6-dimethyl-xanthenone-4 acetic acid (*DMXAA*) is a flavonoid causing DNA damage to endothelial cells that induces apoptosis in preclinical models [126]. When given 1–4 hours after cisplatin chemotherapy, DMXAA or CA-4 induced a markedly increased tumor response in a xenograft model of ovarian carcinoma [129].

The differences between normal and tumor vessels can also be exploited to selectively deliver chemotherapeutic drugs to the tumor vasculature. Peptides containing the asparagines-glycine-arginine (*NGR*) motif, which binds to a specific isoform of CD13 exclusively found in angiogenic vessels, have been used to deliver various antitumor compounds to the tumor vasculature [130]. Targeted liposomal doxorubicin (TVT-DOX) is a form of ligand-targeted nanomedicine that contains the NGR motif on its surface. In a murine xenograft of doxorubicin-resistant ovarian cancer, it was able to more effectively kill angiogenic tumor blood vessels and indirectly the tumor cells that these vessels support than an untargeted formulation of doxorubicin [131].

## 4. Biomarkers

### 4.1. Classical Markers

*Plasmatic CA125* concentration is routinely used in clinical practice as a surrogate marker for clinical response of ovarian cancer treatment [132]. However, CA125 has not been validated in the context of targeted therapies. The mechanism regulating the production and/or secretion of mucin MUC16, which is recognized by the OC125 antibody, is as yet unknown, and it could potentially be altered by biochemical modulation of the tumor [133]. Moreover, in a phase II study of patients receiving BEV and SOR, the authors found a poor concordance between CA125 changes and objective imaging (67% concordance) [134], raising the question whether CA125 monitoring can be used to monitor tumor response to antiangiogenic therapy. 

Response Evaluation Criteria in Solid Tumors (*RECIST*) are routinely used to assess tumor response [135]. They can however not be considered entirely reliable in the context of agents that reduce tumor blood flow because changes in blood flow may precede changes in tumor size [136].

### 4.2. Markers of Angiogenesis

There is a clear correlation in ovarian cancer between markers of angiogenesis and poor *prognosis*. Increased angiogenesis can be identified in various ways.

*Microvessel density* evaluated by the specific endothelial cell marker CD34 is correlated with poor prognosis in ovarian cancer [137, 138]. The Chalkley count with CD34 immunostaining is the most validated method of microvessel density determination [139].

In small retrospective analyses of ovarian tumor samples after surgery and prior to standard chemotherapy, *overexpression of VEGF* as detected by immunohistochemistry on tumor tissue was present in up to 48% of samples and was shown to be independently predictive of poor prognosis [140–142]. However, in recent series of 339 primary ovarian cancers, only 7% showed a high expression of VEGF. The use of different antibodies, scoring systems, and cutoff points might explain the discrepancies between studies. In any case, these latest data suggest that the benefit of anti-VEGF therapy might be limited to a small subset of patients [143]. 

*Other markers of angiogenesis are currently under study. Serum VEGF* levels are independent prognostic markers in ovarian cancer patients [144]. *Genetic testing* also showed promising results, as the simultaneous carriage of 3 single nucleotide polymorphisms associated with increased VEGF production was shown to lead to a significantly impaired overall survival [145], while a 34-gene-profile of angiogenesis-related genes was able to predict the overall survival of ovarian cancer patients [146]. Finally, high expression of new tumor vascular markers, like *STC2, EGFL6, and FZD10*, which are specifically expressed by tumors harboring tumor endothelial cells, have been shown to be associated with a significant decrease in disease-free interval [147]. 

Other biomarkers could be used in the future to *predict* the outcome after targeted therapy. IL-8 plays a significant role in mediating human ovarian carcinoma-derived angiogenesis and tumorigenesis [148], probably independently of VEGF [149]. It was recently shown that the *IL-8 A-251T polymorphism *might be a molecular predictor of response to BEV-based chemotherapy in ovarian cancer patients [150]. *pAKT* may serve as a predictor of resistance to imatinib treatment in ovarian cancer cells [151].

### 4.3. Imaging of Angiogenesis

New noninvasive imaging techniques are currently under study. In a retrospective study of 49 women with primary ovarian cancer or metastatic tumors to the ovary, three-dimensional power Doppler *ultrasound* (3D-PDU), which allows tumor vascularization assessment, showed that vascularization was higher in advanced stage and metastatic ovarian cancers than in early stage ovarian cancer [152]. In a retrospective study of 41 women with epithelial ovarian cancer, researchers found that dynamic contrast-enhanced *magnetic resonance* imaging (DCE-MRI) could help distinguish among benign, borderline, and invasive tumors and was correlated with tumoral angiogenic status, specifically the pericyte coverage index and VEGF expression [153]. 

Tracers focusing on VEGF and VEGFR2 have been developed to visualize angiogenesis-related events with noninvasive *positron emission tomography* (PET) imaging [154, 155]. In preclinical murine models of ovarian carcinoma treated with vascular-disrupting agents, [^18^F]FDG PET imaging could predict tumor response as early as 2 hours after therapy [128].

## 5. Conclusion

Antiangiogenic therapy in ovarian cancer is very promising so far, at least in phase II trials. This is probably due to the highly angiogenesis-dependent pathophysiology of this disease. We should however keep in mind that angiogenesis might not be the driving force behind all cases of epithelial ovarian cancer and that we are still missing large placebo-controlled phase III trials that show a benefit in term of PFS or overall survival. Tools to detect the patients that are likely to benefit from antiangiogenic treatment have yet to be validated in the clinic. This would allow us to restrict the use of these very potent but also onerous new drugs to those who are most likely to benefit. A better selection of patients would also help to reduce the high complication rate seen with these agents, in particular GI perforations. The optimal duration of maintenance treatment with BEV will also have to be evaluated, and pharmaco-economic considerations will have to be addressed. Finally, combined targeting of tumor cells, endothelial cells, and pericytes (which play an important role in the stabilization of endothelial cells) is a very interesting approach that warrants further studies.

In conclusion, antivascular treatment for epithelial ovarian cancer is a very promising approach that still needs to be validated in the phase III setting. As many patients as possible should be encouraged to take part in well-designed clinical trials.

## Figures and Tables

**Table 1:** Ongoing studies with bevacizumab (BEV) in ovarian cancer.

Stage of the disease	Phase	Intervention	Trial number
Monotherapy			

Recurrence after prior therapy with maintenance BEV	II	BEV monotherapy	NCT00866723

Combination with chemotherapy			

Newly diagnosed	III	Carboplatin and paclitaxel with versus without BEV	ICON7NCT00483782
Previously untreated stage III or IV	III	Carboplatin and paclitaxel versus carboplatin, paclitaxel, and concurrent BEV with versus without extended BEV	GOG218NCT00262847
Adjuvant	II	Carboplatin, paclitaxel and BEV (BEV omitted in first cycle)	OVCANCT00129727
Newly diagnosed stage III/IV	II	Carboplatin, paclitaxel and BEV	AV53206sNCT00127920
Newly diagnosed stage IB-IV	II	Oxaliplatin and docetaxel with BEV	TEACONCT00296816
Newly diagnosed stage II-III	II	IV paclitaxel, IP cisplatin and IV BEV followed by BEV consolidation	AVF3953NCT00511992
Initial treatment of optimal stage II or III (adjuvant)	II	IV and IP paclitaxel, IP cisplatin, and IV BEV	06-064NCT00588237
Platinum-sensitive recurrent	III	Carboplatin and paclitaxel with versus without BEV followed by secondary cytoreduction surgery	GOG213NCT00565851
Platinum-sensitive recurrent	III	Carboplatin and gemcitabine with versus without BEV	AVF4095gNCT00434642
Platinum-sensitive recurrent	II	Gemcitabine, carboplatin and BEV	2005CO073NCT00267696
Platinum-sensitive recurrent	II	Carboplatin and liposomal doxorubicin plus BEV	CR015094NCT00698451
Platinum-sensitive recurrent	II	Oxaliplatin, gemcitabine, and BEV	DF 04-356NCT00418093
Recurrent having failed platinum- and taxane-based regimens	II	Pemetrexed and BEV	08-0508NCT00868192
Platinum-resistant recurrent	II	Weekly topotecan with BEV	AVF3648sNCT00343044
Platinum-resistant recurrent	II	BEV and docetaxel	MCC-14920NCT00504257
Platinum-resistant recurrent	II	BEV and carboplatin	2008-000878-20NCT00744718
Platinum-resistant recurrent	II	BEV and liposomal doxorubicin	AVF3910sNCT00846612
Platinum-resistant recurrent	II	Sequential BEV and metronomic cyclophosphamide	08-148NCT00856180
Platinum-resistant recurrent	II	BEV and albumin-bound paclitaxel	ALSSOPR0501NCT00407563
2nd or later complete remission, or untreated or refractory to platinum treatment or no response to salvage treatment	II	Stem-cell transplant trial evaluating treatment with BEV plus gemcitabine, docetaxel, melphalan, and carboplatin	2007-0368NCT00583622
Advanced peritoneal carcinomatosis	I	IP oxaliplatin and paclitaxel plus IV paclitaxel and BEV	2006-1068NCT00491855

Combination with other targeted therapies			

Newly diagnosed	II	BEV and erlotinib as 1st line consolidation chemo after carboplatin, paclitaxel, and BEV induction therapy	07-039NCT00520013
Relapsed or refractory	II	BEV and erlotinib	UARIZ-05-0178-01NCT00696670
Recurrent or metastatic	II	BEV and erlotinib	NCI-6759NCT00126542
Refractory or recurrent	II	BEV and sorafenib	NCI-07-C-0058NCT00436215
Persistent or recurrent	II	BEV with or without everolimus	GOG-0186GNCT00886691

Studies were accessed from http://www.clinicaltrials.gov/ on May 17, 2009

IV = intravenous, IP = intraperitoneal

**Table 2:** Ongoing trials with multitargeted tyrosine kinase inhibitors in ovarian cancer.

Agent	Targets	Phase	Intervention	Stage of the disease	Trial number
Sunitinib	VEGFRPDGFRc-kitRETCSF-1Rflt-3	II	Sunitinib monotherapy	Platinum-resistant recurrent	AGO-OVAR 2.11
					NCT00543049
					(Germany)
		II	Sunitinib monotherapy	Recurrent or refractory	DF08-056
					NCT00768144
					(United States)
		II	Sunitinib monotherapy	Advanced and/or metastatic	CAN-NCIC-IND185
					NCT00388037
					(Canada)

Sorafenib	Raf-1VEGFRPDGFRBflt-3c-kit	II	Sorafenib maintenance versus placebo	CR after standard platinum therapy	NCT00791778
		II	Paclitaxel and carboplatin +/− sorafenib	1st line	SCRI GYN 19NCT00390611
		II	Paclitaxel and carboplatin +/− sorafenib	Platinum-sensitive recurrent	CASE-CWRU-2804NCT00096200
		II	Topotecan + sorafenib	Platinum-resistant recurrent	GYN06-111NCT00526799

Pazopanib	VEGFRPDGFRc-kit	III	Pazopanib maintenance versus placebo	After 1st line chemo	AGO-OVAR16NCT00866697
		II	Pazopanib monotherapy	Recurrent	VEG104450NCT00281632
		I	Metronomic topotecan + pazopanib	Persistent or recurrent	NCT00800345

XL999	VEGFRPDGFRFGFRflt-3Src	II	XL999 monotherapy	Recurrent	NCT00277290

Motesanib	VEGFRPDGFRc-kit	II	Motesanib monotherapy	Persistent or recurrent	NCT00574951

Vandetanib	VEGFREGFR	II	Docetaxel +/− vandetanib	Persistent or recurrent	SWOG-S0904NCT00872989
		I/II	Pegliposomal doxorubicin +/− vandetanib	Platinum-refractory recurrent	NCT00862836

Studies were accessed from http://www.clinicaltrials.gov/ on May 17, 2009

VEGFR = vascular endothelial growth factor receptor, PDGFR = platelet-derived growth factor receptor,
